# Resveratrol ameliorates aging-related metabolic phenotypes by inhibiting cAMP phosphodiesterases

**DOI:** 10.1186/1753-6561-6-S3-P73

**Published:** 2012-06-27

**Authors:** Sung-Jun Park, Faiyaz Ahmad, Andrew Philp, Keith Baar, Tishan Williams, Haibin Luo, Hengming Ke, Holger Rehmann, Ronald Taussig, Alexandra L Brown, Myung K Kim, Michael A Beaven, Alex B Burgin, Vincent Manganiello, Jay H Chung

**Affiliations:** 1 Laboratory of Obesity and Aging Research, Genetics and Developmental Biology Center, National Heart Lung and Blood Institute, National Institutes of Health, Bethesda, MD 20892, USA; 2 Cardiovascular Pulmonary Branch, National Heart Lung and Blood Institute, National Institutes of Health, Bethesda, MD 20892, USA; 3 Laboratory of Molecular Immunology, National Heart Lung and Blood Institute, National Institutes of Health, Bethesda, MD 20892, USA; 4 Functional Molecular Biology Laboratory, University of California Davis, Davis, CA 95616, USA; 5 Department of Biochemistry and Biophysics, The University of North Carolina, Chapel Hill, NC 27599-7260, USA; 6 Structural Biology Lab, School of Pharmaceutical Sciences, Sun Yat-sen University, Guangzhou, 510006, P. R. China; 7 Department of Molecular Cancer Research, Centre for Biomedical Genetics and Cancer Genomics Centre, University Medical Center, 3584 CG Utrecht, The Netherlands; 8 Department of Pharmacology, University of Texas Southwestern Medical Center, Dallas, TX 75390, USA; 9 Emerald BioStructures, 7869 NE Day Road West, Bainbridge Island, WA 98110, USA

## 

Resveratrol, a polyphenol in red wine, has been reported as a calorie restriction mimetic with potential antiaging and antidiabetogenic properties. It is widely consumed as a nutritional supplement, but its mechanism of action remains a mystery. Here, we report that the metabolic effects of resveratrol result from competitive inhibition of cAMP-degrading phosphodiesterases, leading to elevated cAMP levels. The resulting activation of Epac1, a cAMP effector protein, increases intracellular Ca2^+^ levels and activates the CamKKβ-AMPK pathway via phospholipase C and the ryanodine receptor Ca2^+^-release channel. As a consequence, resveratrol increases NAD+ and the activity of Sirt1. Inhibiting PDE4 with rolipram reproduces all of the metabolic benefits of resveratrol, including prevention of diet-induced obesity and an increase in mitochondrial function, physical stamina, and glucose tolerance in mice.

